# Supplementary material for: Structural Phase Transitions and Thermal Degradation Process of MAPbCl3 Single Crystals Studied by Raman and Brillouin Scattering
Source: Materials (Basel). 2022 Nov 17;15(22):8151. doi: 10.3390/ma15228151 (PMC9699233; doi:10.3390/ma15228151)
Supplement: Supplementary file 1 [file materials-15-08151-s001.zip › materials-1981969-supplementary.pdf]

Supplementary Materials

Structural Phase Transitions and Thermal Degradation Process of $MAPbCl_3$ Single Crystals Studied by Raman and Brillouin Scattering

Furqanul Hassan Naqvi, Jae-Hyeon Ko

School of Nano Convergence Technology, Nano Convergence Technology Center, Hallym University, Chuncheon, Gangwondo 24252, Republic of Korea

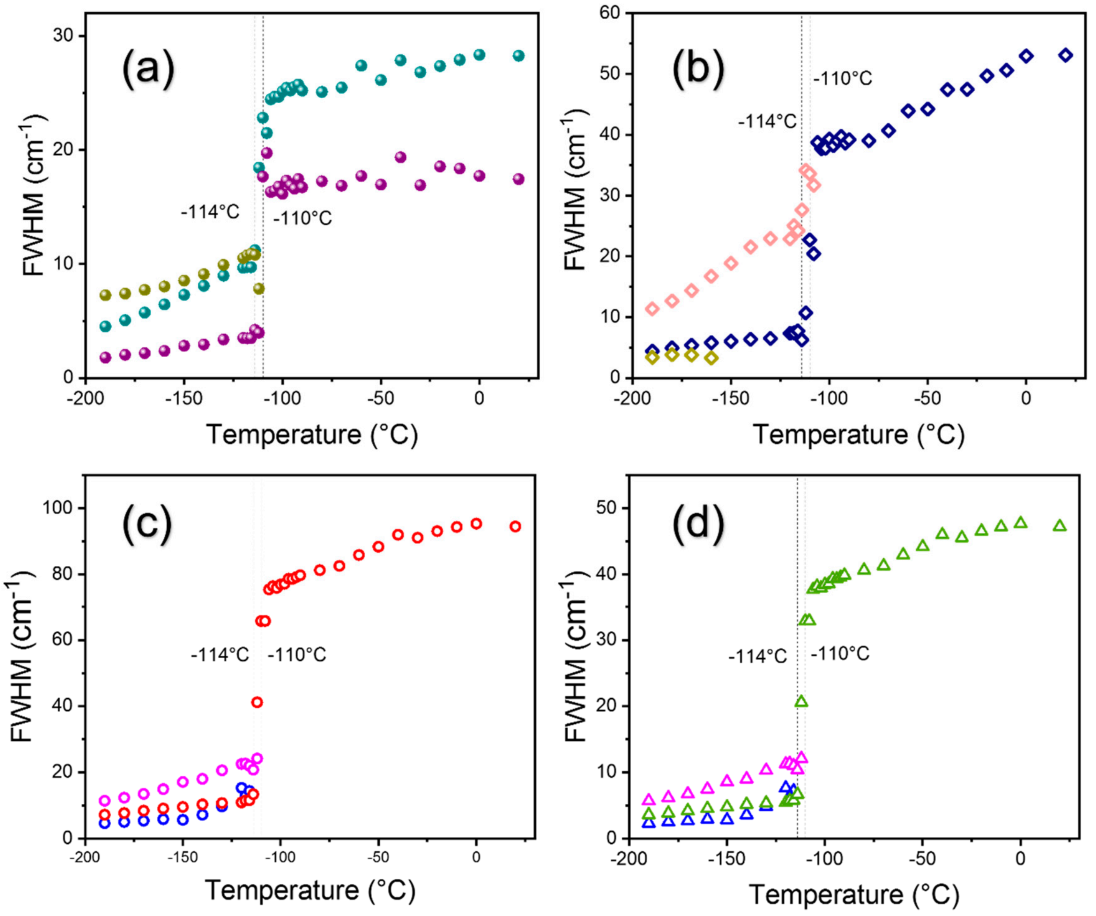

Figure S1: Temperature dependences of the full width at half maximum (FWHM) in 10-300 cm^{-1} frequency range (a-d). Phase boundaries are shown as vertical dotted lines along with the phase transition temperatures

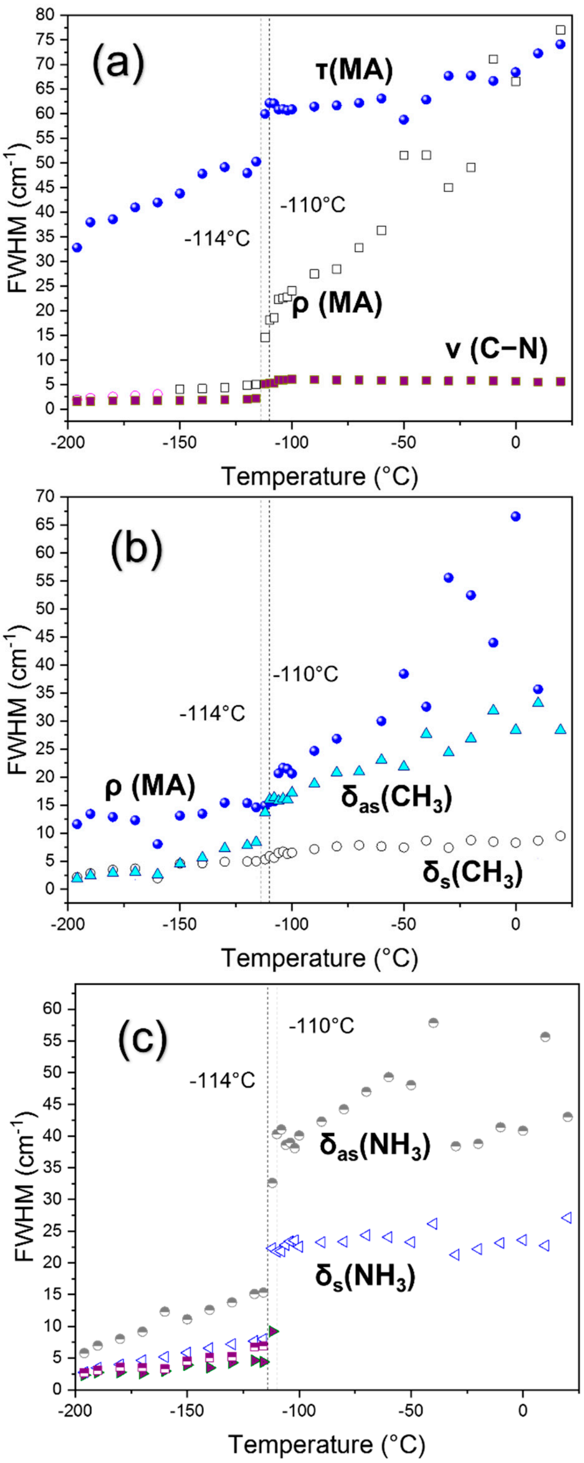

Figure S2: Temperature dependences of the full width at half maximum (FWHM) in 400-1700 cm^{-1} frequency range (a-c). Phase boundaries are shown as vertical dotted lines along with the phase transition temperatures.

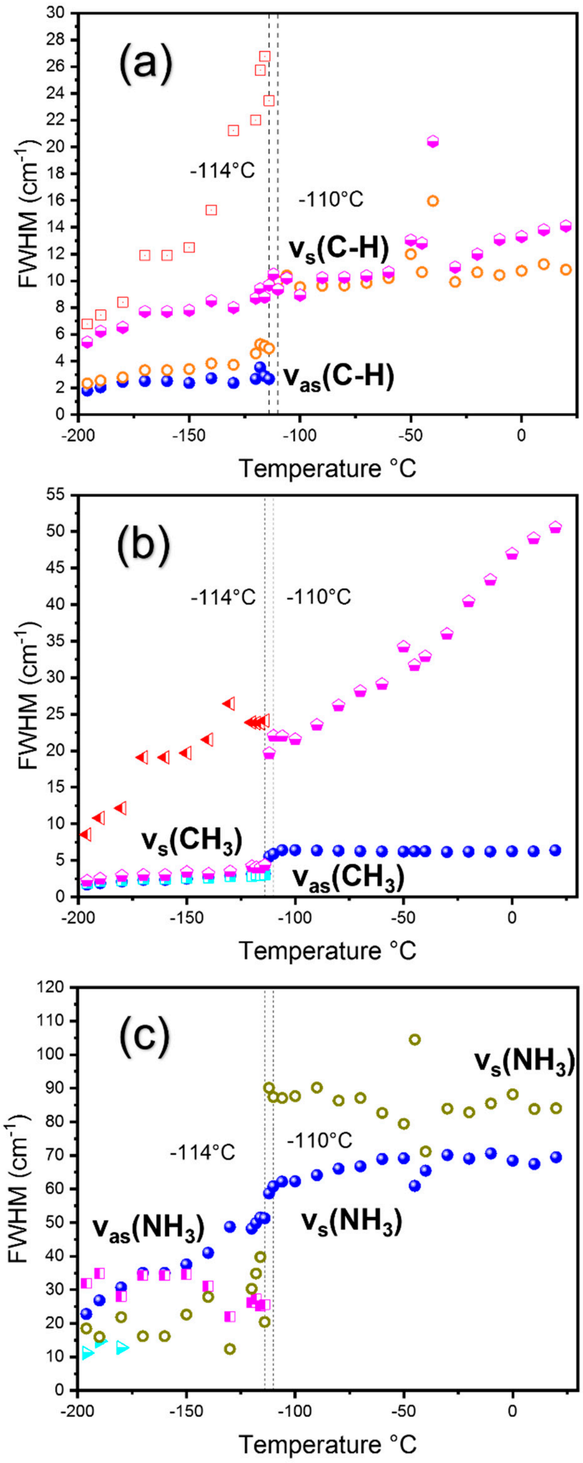

Figure S3: Temperature dependences of the full width at half maximum (FWHM) in 2700-3400 cm^{-1} frequency range (a-c). Phase boundaries are shown as vertical dotted lines along with the phase transition temperatures.

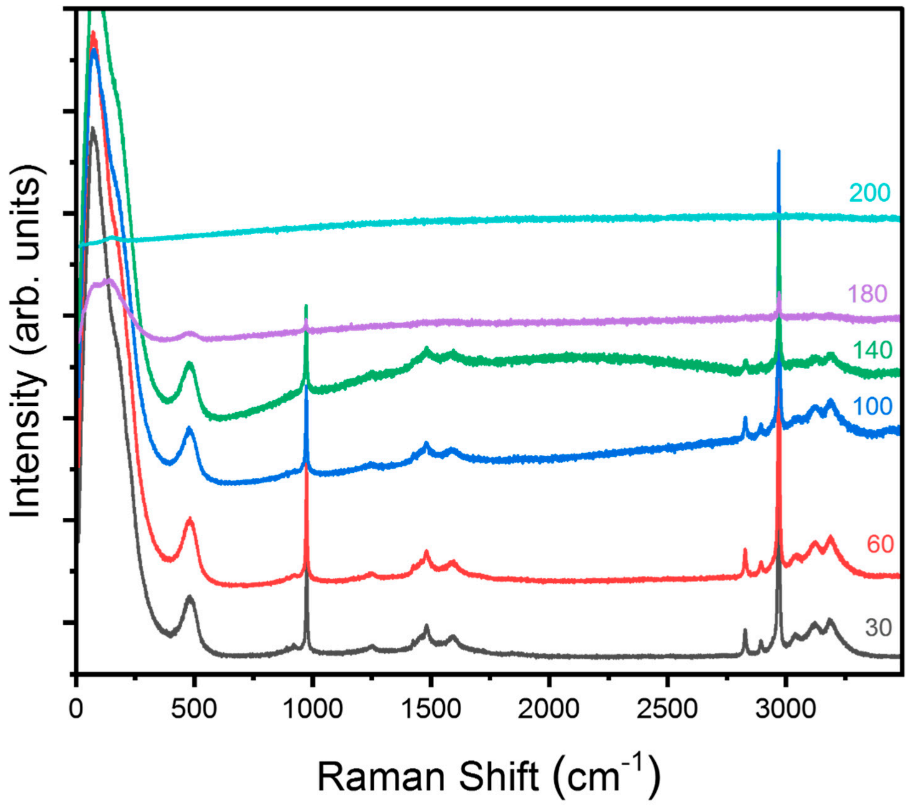

Figure S4: Raman spectra of $MAPbCl_3$ in a wide frequency range of 10-3500 cm^{-1} recorded in the temperature range from room temperature (RT) to 200°C.

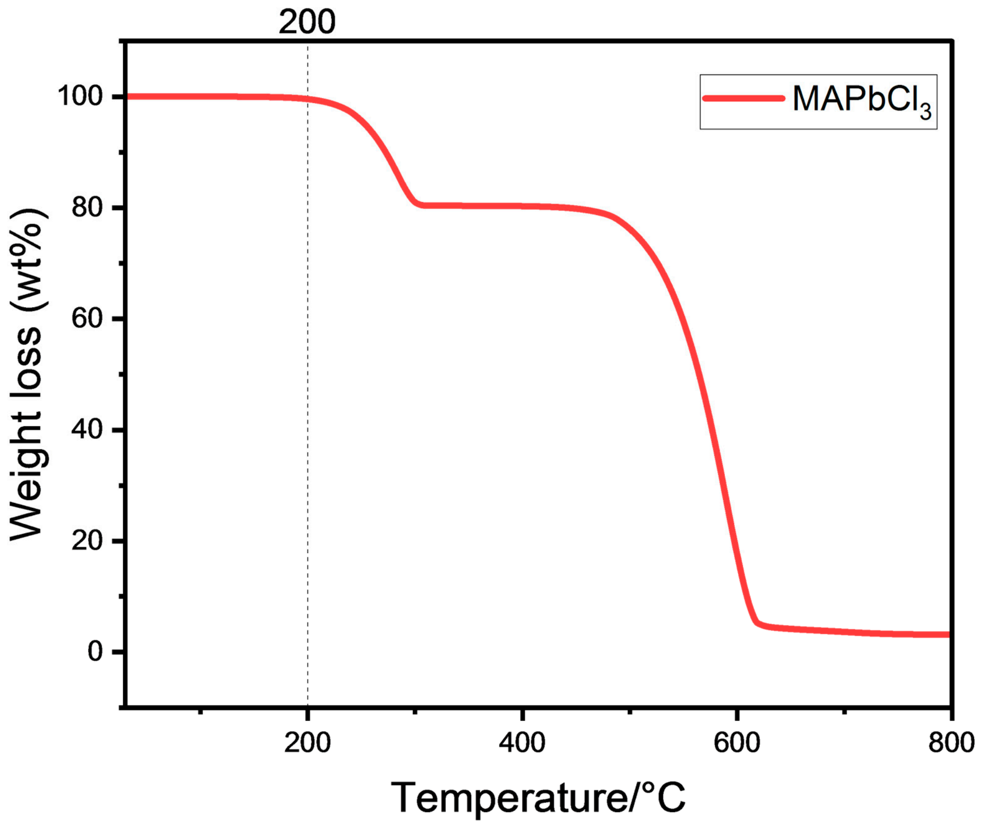

Figure S5. Thermogravimetric analysis curve for synthesized $MAPbCl_3$ crystal.

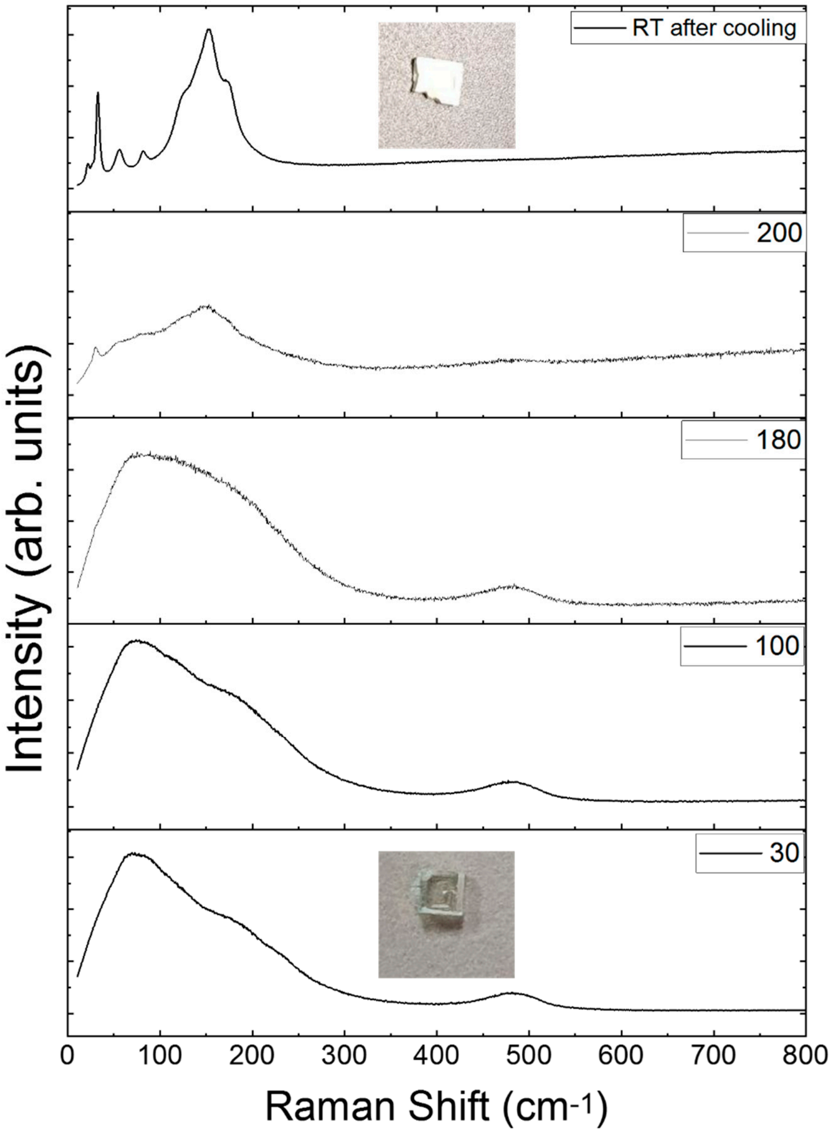

Figure S6: Raman spectra of $MAPbCl_3$ presented in a limited frequency range of 10-800 cm^{-1} from room temperature (RT) to 200°C. Inset figures show the single crystal before (lower figure) and after (upper figure) the degradation.

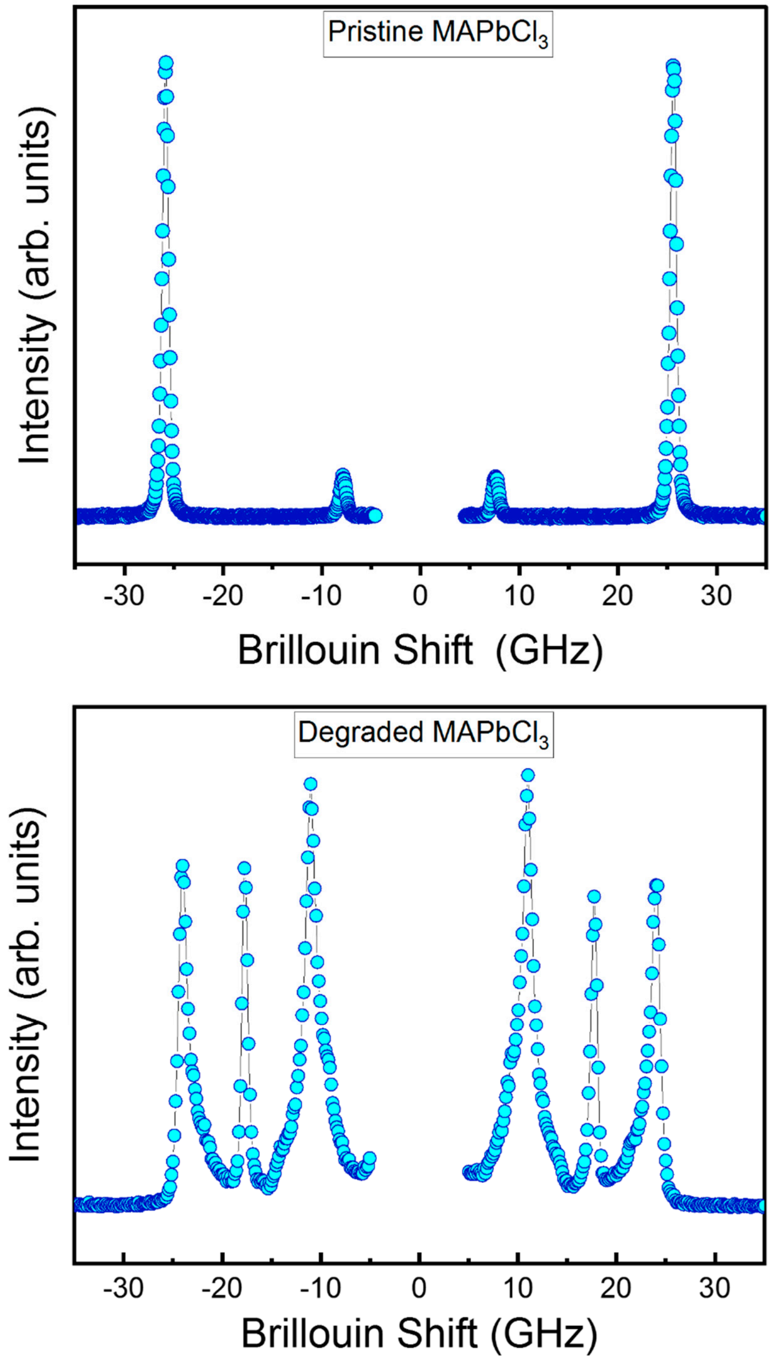

Figure S7: Comparison of Brillouin spectra of the pristine $MAPbCl_3$ single crystal (a) with the crystal kept for one month in the ambient condition (b)
